# Happiness and high reliability develop affective trust in in-vehicle agents

**DOI:** 10.3389/fpsyg.2023.1129294

**Published:** 2023-03-14

**Authors:** Scott Zieger, Jiayuan Dong, Skye Taylor, Caitlyn Sanford, Myounghoon Jeon

**Affiliations:** ^1^Mind Music Machine Lab, Department of Industrial and Systems Engineering, Virginia Polytechnic Institute and State University, Blacksburg, VA, United States; ^2^Link Lab, Department of Engineering Systems and Environment, University of Virginia, Charlottesville, VA, United States

**Keywords:** conditionally automated vehicles, in-vehicle agents, reliability, emotions, trust

## Abstract

The advancement of Conditionally Automated Vehicles (CAVs) requires research into critical factors to achieve an optimal interaction between drivers and vehicles. The present study investigated the impact of driver emotions and in-vehicle agent (IVA) reliability on drivers’ perceptions, trust, perceived workload, situation awareness (SA), and driving performance toward a Level 3 automated vehicle system. Two humanoid robots acted as the in-vehicle intelligent agents to guide and communicate with the drivers during the experiment. Forty-eight college students participated in the driving simulator study. The participants each experienced a 12-min writing task to induce their designated emotion (happy, angry, or neutral) prior to the driving task. Their affective states were measured before the induction, after the induction, and after the experiment by completing an emotion assessment questionnaire. During the driving scenarios, IVAs informed the participants about five upcoming driving events and three of them asked for the participants to take over control. Participants’ SA and takeover driving performance were measured during driving; in addition, participants reported their subjective judgment ratings, trust, and perceived workload (NASA-TLX) toward the Level 3 automated vehicle system after each driving scenario. The results suggested that there was an interaction between emotions and agent reliability contributing to the part of affective trust and the jerk rate in takeover performance. Participants in the happy and high reliability conditions were shown to have a higher affective trust and a lower jerk rate than other emotions in the low reliability condition; however, no significant difference was found in the cognitive trust and other driving performance measures. We suggested that affective trust can be achieved only when both conditions met, including drivers’ happy emotion and high reliability. Happy participants also perceived more physical demand than angry and neutral participants. Our results indicated that trust depends on driver emotional states interacting with reliability of the system, which suggested future research and design should consider the impact of driver emotions and system reliability on automated vehicles.

## 1. Introduction

Conditionally Automated Vehicles (CAVs) are a developing technology that will greatly impact transportation in the future. With these systems, drivers will need to rely on the judgment of artificial intelligence to make the safest decisions possible, particularly at Level 3 automation and beyond where the vehicle has primary control (SAE, 2018). Trust, in the context of CAVs, is defined as the “attitude that an agent will help achieve an individual’s goals in a situation characterized by uncertainty and vulnerability” (Lee and See, 2004). Trust, in this case, is critically important to ensure proper use of these systems to their greatest extent. Previous studies have identified that the emotions of a user can have a significant influence on trust development (Dunn and Schweitzer, 2005) and on driving performance (Jeon et al., 2014a,b; Jeon, 2016; Sterkenburg and Jeon, 2020). However, few studies have investigated how these emotions influence trust in a CAV context. Cognitive appraisal determines how individuals evaluate emotional situations. According to Smith and Ellsworth (1985), emotions have been categorized with different patterns of cognitive appraisals, especially the self-other responsibility control significantly influences people’s trust (Dunn and Schweitzer, 2005). For example, anger has a high other responsibility control as an angry person perceives other people to be responsible for unpleasant situations. Emotions are also categorized with other dimensions, such as certainty and attentional activities (Smith and Ellsworth, 1985). Therefore, in the present study, we decided to induce happy, angry, or neutral (baseline) emotional states on the participants to observe this potential relationship between cognitive appraisals in different emotions and trust toward automated vehicle systems.

The use of in-vehicle agents (IVAs) plays a critical role in communication with drivers in CAVs. These IVAs are “anthropomorphized intelligent systems that can interact with drivers using natural human language” (Lee and Jeon, 2022). Due to the current technology, these can vary in reliability which, along with emotions, can have a strong impact on the effectiveness of driver-agent interaction (Lee and Jeon, 2022). To investigate the influence of reliability of IVAs, we created two separate reliability levels based on the percentage of correctly presented information: high (100% reliability) and low (67% reliability) conditions. In the present study, we focused on the impact of drivers’ emotions and IVA’s reliability level on drivers’ situation awareness (SA), perceptions, trust, perceived workload, and driving performance toward a Level 3 automated vehicle system.

## 2. Related work

### 2.1. Emotion induction

Multiple methods of emotion induction have been used in previous studies. One of these was a priming task, defined as a method of manipulation in which individuals were asked to recall a time that they felt an emotion without providing further elaboration. This was determined by Dunn and Schweitzer (2005) to have no significant emotional effect. In the present study, the desired emotion was induced using an Autobiographical Emotional Memory Task (AEMT). As described by FakhrHosseini and Jeon (2017), in this method of induction, the participant writes for 12 min about one or more of their past experiences related to the emotion being induced (e.g., happy or angry). The participant is asked to immerse themselves in the memory of their experience, writing as clearly as possible. This type of writing task has demonstrated an effective method of inducing the intended emotion and does not require additional technologies needed for music or film induction methods (Mills and D'Mello, 2014).

### 2.2. Agent anthropomorphism

The anthropomorphism of a robotic agent, defined as “a process of inductive inference whereby people attribute to nonhumans distinctively human characteristics” (Waytz et al., 2014), has shown to significantly influence user trust, particularly in automated vehicles. Drivers in a vehicle with humanlike characteristics (such as a name, gender, and a voice) had a higher physiological trust than those in a non-anthropomorphic vehicle (Waytz et al., 2014). Physiological trust indicated the participants’ level of relaxation in an accident scenario. This was determined through changes in heart rate, measured using electrocardiography, and startle, measured using a 0 to 10 scale by 42 independent raters. Self-reported trust, however, did not differ significantly (Waytz et al., 2014). Furthermore, in unavoidable accidents, automated vehicles were blamed significantly more than non-automated vehicles (Waytz et al., 2014). However, drivers placed significantly less blame on vehicles with anthropomorphic characteristics (Waytz et al., 2014). A previous study also showed that drivers preferred embodied agents over voice agents because they found the humanoid robots as more likeable and warmer (Wang et al., 2021).

Beyond a humanlike appearance, changes in an agent’s voice have also shown significant effects on driver trust and behavior. In both manual and automated driving, the dominance of the voice caused significant changes in situation awareness (SA). The SA of a manual driver increased when the agent had a more dominant voice; however, this was reversed in Level 3 automated driving, where a more submissive voice increased SA (Yoo et al., 2022). Additionally, drivers in an automated vehicle with a submissive voice demonstrated a higher level of trust and improvements in regulating angry emotions (Yoo et al., 2022). Agents with speech patterns designed to improve SA were effective in increasing SA and performance of angry drivers (Jeon et al., 2015). These SA speech patterns were suggestive/notification style prompts that would ask or comment about the driver’s surroundings (“If you see any restaurant, let me know.”). These agents were also viewed as more likable than those with directive/command style speech patterns designed to regulate emotions (“Forget your angry feelings. You are driving now.”).

While our study did not investigate the interactions between anthropomorphism and the other variables, it is important to understand the effects that it might have. The present study involved two humanoid robots, NAO and Milo, playing the roles of IVAs that communicate with the drivers. Each IVA’s name was told to the participants. Note that we did not manipulate the degree of embodiment of the two robots as a study variable; instead, the two robots were used to represent different levels of reliability, respectively. To ensure consistency and minimize the plausible robot effects, the mapping of reliability on each robot agent was counterbalanced across participants.

### 2.3. Trust

#### 2.3.1. Agent trust

Beyond elements designed to humanize an agent, there were also other factors that influenced user trust in the system. According to Koo et al. (2015), IVAs can inform the user of autonomous actions in different ways. This can be through a message that explains the context of why an action was taken, how the action will be accomplished, or a combined explanation of both. While the combined “how and why” message was the safest method in terms of driving behavior and steering control, it also created the highest level of anxiety out of the three due to a possible increase in cognitive load (Koo et al., 2015). “Why” only messages, on the other hand, created the highest trust and lowest anxiety levels (Koo et al., 2015). In situations where safety is not a critical issue, “why” only messages may be preferred to build an acceptable level of trust (Koo et al., 2015), which we also considered in the present study.

Another method of increasing trust lies in the time spent using the system. With both initially trusting and distrusting drivers, 10 min in the simulator experiencing the sounds, environment, and system of highly automated driving (HAD) before the study significantly increased the level of trust in HAD (Manchon et al., 2022). After this time, drivers were shown to monitor the road with fewer glances and with more time spent engaging in Non-Driving Related Activities (NDRA). Additionally, this had a greater effect on drivers who were initially distrusting the system, as their level of trust development increased significantly more.

#### 2.3.2. Cognitive and affective trust

Developing trust between drivers and automated vehicles has been a challenge to researchers. In addition to using the Trust in Automation scale (Jian et al., 2000), we also desired to measure other types of trust toward a Level 3 automated vehicle system. According to McAllister (1995), cognitive (cognition-based) trust is defined as trust based on the knowledge and evidence on someone’s ability and achievements; affective (affect-based) trust is defined as trust based on the emotional bond with someone. Cognitive and affective trust could be a considerable factor in improving workers’ performance for cooperative organizations (Morrow et al., 2004; Johnson and Grayson, 2005) and impact users’ satisfaction and loyalty (Trif, 2013). There are various automated driving research studies investigating trust in automation but none in terms of cognitive and affective trust. The present study involved a trust scale including cognitive and affective trust (McAllister, 1995) to determine any correlation among drivers’ emotions, reliability, and trust in automated driving systems.

#### 2.3.3. The relationship between emotion and trust

The emotional state of an individual has a large influence on driving behavior. Jeon et al. (2014a,b) found that angry and happy drivers had a greater number of errors than drivers who were fearful or emotionally neutral. Angry drivers were also shown to have the lowest level of perceived safety, and happier drivers were shown to have the highest perceived workload. Happy people typically want to maintain their happiness (Isen, 1987; Wegener et al., 1995), but a challenging driving task might have served as an obstacle to it, which made them perceive relatively high workload.

In previous studies, emotion was shown to have a large effect on the levels of trust. According to Dunn and Schweitzer (2005), when the trustee was unfamiliar, happy individuals had significantly higher levels of trust in the trustee than sad individuals, and sad individuals had higher levels of trust in the trustee than angry individuals. If the participant was familiar with the trustee, emotions had no significant effect on trust in the trustee (Dunn and Schweitzer, 2005). Inversely, the level of trust in an automated vehicle was also shown to influence emotional state. According to a study conducted by Dixon et al. (2020), drivers who gained trust in the automated vehicle were significantly more likely to display a happy emotion. On the other hand, a decrease in trust was correlated with displays of an angry emotion.

Trust is a crucial predictor of people’s willingness to engage with technologies (Plaks et al., 2022). Literature also shows that emotions are important factors influencing trust toward automated systems (Cho et al., 2015; Granatyr et al., 2017). Therefore, the knowledge of emotional effects on automation trust is a matter of critical importance in the design of trustable automated systems. Through the use of emotion induction, we specifically focused on the influence of emotional states on driver trust. The findings made by Dunn and Schweitzer (2005) are particularly comprehensive, factoring in both the role of familiarity of the trustee and the influence of personal emotion. Given that our agent may be considered “unfamiliar” to participants, we expect to see similar results.

#### 2.3.4. The relationship between reliability and trust

A system’s reliability could directly affect the user’s trust in the system. Muir and Moray (1996) proposed that faith was the primary contributor to trust. However, a replication study conducted by Long et al. (2022) falsified this finding two decades later and showed reliability to be the best predictor of trust over faith. Given that reliability was shown to be a higher predictor of trust (Long et al., 2022), we believe that investigating the impact of reliability on trust will yield more significant results than the impact of faith on trust.

Reliability also impacts the driver’s experience and decisions. In the study by Chancey et al. (2017), participants were more likely to comply with the system if they had higher trust in the system. Therefore, we expect that agents who are more reliable may lead to a higher level of trust from a participant. With regards to the emotional state of the driver, low reliability resulted in more negative emotions and high reliability resulted in more positive emotions (Fahim et al., 2021). In addition, a more reliable agent has been shown to reduce anxiety but was not shown to lower hostility or loneliness (Fahim et al., 2021). We expect these results to be similar to those found by Chancey et al. (2017), with a correlation between the level of trust and the reliability of the agent. However, with the addition of emotional states, there may be a significant interaction that changes these results. Given that this will likely be the first interaction with automated vehicles for many of our participants, we predicted that trust levels would improve throughout the study regardless of the reliability of the system. We minimized this by counterbalancing the order of reliable and unreliable agents between participants in the present study.

#### 2.3.5. Research gap and unique contributions

Previous research focused heavily on the connection between trust and factors of reliability. However, the amount of research connecting emotion and trust is limited in the automated vehicle context. Additionally, there is a lack of information about the influences of emotion on both cognitive trust and affective trust. This paper intends to expand on these factors, as well as determine an interaction between emotion and reliability on trust. With the findings in this paper, we aim to provide contributions to future CAV designs, namely regarding the design and implementation of IVAs.

In the current study, our objective was to investigate how drivers’ emotional states and the reliability of IVAs influence driver response in a Level 3 automated vehicle. To this end, we had the following research questions:

- RQ1: How do drivers’ emotions and the in-vehicle agent’s reliability impact drivers’ subjective judgments (Godspeed, Social Presence, RoSAS, and SASSI), situation awareness (SAGAT), and trust (Trust in Automation, Cognitive and Affective Trust) on a Level 3 automated vehicle?
- RQ2: How does the interaction between drivers’ emotional states and the in-vehicle agent’s reliability influence drivers’ trust (Trust in Automation, Cognitive and Affective Trust) on a Level 3 automated vehicle?

## 3. Method

### 3.1. Participants

Forty-eight participants (33 male, 14 female, 1 non-binary) were recruited. Two participants were excluded from the study due to simulator sickness and were not counted in the total 48. All participants were between the ages of 15 and 30, had an active drivers’ license, and had normal or corrected-to-normal vision and hearing. The average age was 21.1 (SD = 2.0). They had an average driving experience of 4.6 years (SD = 2.1), drove an average of 7.2 times a week (SD = 2.3) and an average of 7254.8 miles per year (SD = 10478.3).

### 3.2. Stimuli and equipment

**Figure 1:**
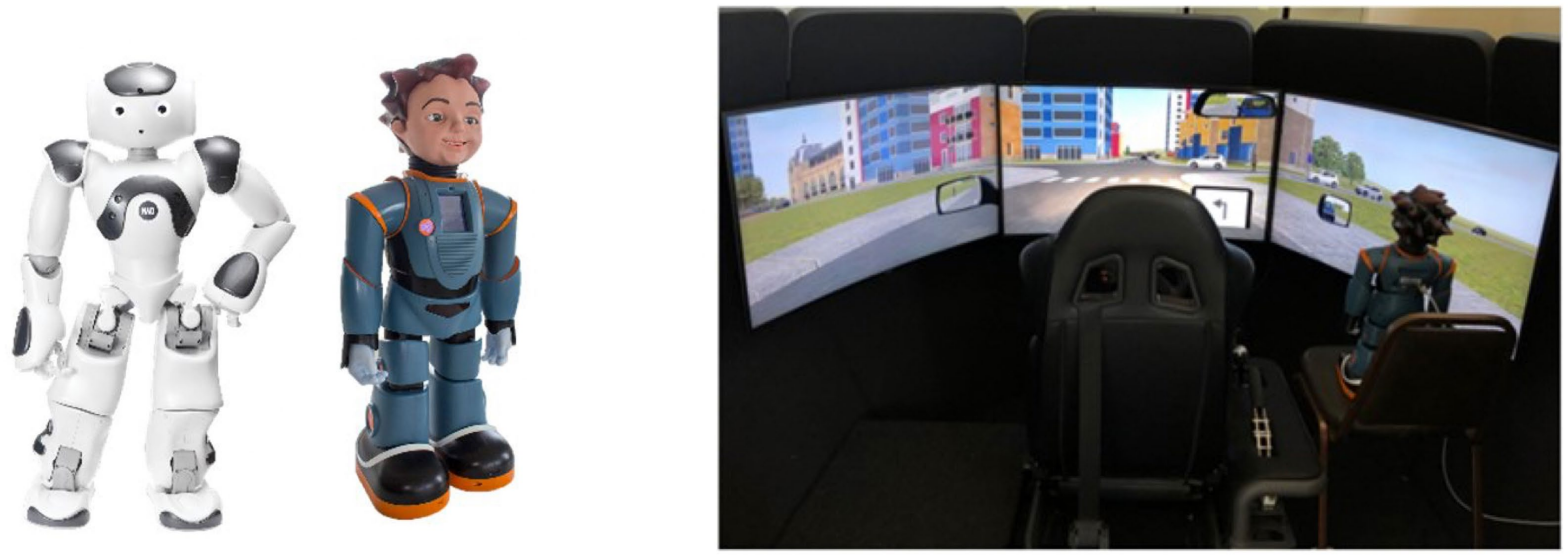
Robotic IVAs NAO and Milo (left) and Simulator and agent setup (right). NAO is placed in the same position when used.

Participants used a *Nervtech* driving simulator which included a steering wheel, gas and brake pedals, and an adjustable seat, and three visual displays providing a horizontal view of 120°. Between and during each trial, desktop computer and tablet were used to complete a series of surveys. IVAs were represented using two programmable humanoid robots, with connection to WiFi and Bluetooth. The first was NAO (Figure 1; Height: 22.6 in) by *SoftBank Robotics*, and the second was Milo (Figure 1; Height: 24 in) by *RoboKind.* These robots were placed next to the participant for the duration of each trial as displayed by Figure 1 (right) and were used to communicate driving and vehicle information.

IVAs’ speech clips were created through Amazon polly TTS (text-to-speech) service. Agent reliability was counterbalanced across participants to reduce the effects of robot appearance. For half of the participants, Milo represented a highly reliable agent and NAO represented an unreliable agent. This was reversed for the other half. SCANeR Studio, developed by *AV Simulation*, was used to develop driving scenarios. The computer used for this software contained an i7-8086K CPU and a Nvidia GTX 1080 graphics card.

### 3.3. Experimental design

The study used a 2×3 mixed factorial design with reliability as a within-subjects variable (high vs. low reliability) and emotion as a between-subjects variable (happy, angry, and neutral emotions). Sixteen participants experienced the happy emotion condition (13 male, 2 female, 1 non-binary), 16 experienced the angry emotion condition (10 male, 6 female), and 16 experienced the neutral emotion condition (10 male, 6 female). The reliability levels of IVAs were defined by the accuracy of information provided by the IVAs for each takeover event. Table 1 includes the scripts or instructions of the agent’s speech for each takeover event during each driving scenario. Each agent’s instruction was divided by three distinct pieces of information regarding the takeover event. In the high reliability condition, IVAs provided drivers with information with 100% reliability; however, in the low reliability condition, IVAs provided drivers information with 67% reliability (one out of three pieces of information is wrong). Each reliability condition was experienced, in fully counterbalanced order, by each participant with three optional takeover events per reliability condition. Optional takeover events allowed the drivers to choose whether to follow the agent’s instruction at each takeover event, therefore, served as the compliance to the agent’s instruction. The takeover events included a blockage on the road, a hardware or mechanical error, and hazardous weather including rain and fog. The two scenarios used the same city and events; however, the driving route and order of events differed. For example, the route of the first scenario started with driving on a straight road, but the second scenario started with a turning signal ahead. Regarding the differences in the order of events, the second event of the first scenario is car swerving, whereas this event is the fifth event of the second scenario. These routes contained both straight and curved roadways, traffic signals, intersections, and other vehicles driving on the road.

**Table 1:** Driving scenario agent’s scripts.

Scenario 1 events	[Reliable/Unreliable]	Scenario 2 events	[Reliable/Unreliable]
Construction site	Please take over (1). The vehicle's front cameras detect an obstacle (2) [around a quarter mile/3 miles] ahead (3).	Construction Site	Please take over (1). The vehicle's front cameras detect an obstacle (2) [700 feet/2 miles] ahead(3).
Car swerves	The car in front of you (1) is expected to swerve into your lane in [1000 feet/2 miles] (2) based on the system's prediction program (3).	Car Swerves	The vehicle to your [left/right] (1) is expected to swerve into your lane (2) based on the system's decision model (3).
Decision error	Please take over (1). There is an error (2) in the system's decision-making code (3). …Nevermind	Sensor Malfunction	Please take over (1). The front right sensor is malfunctioning (2) Based on the test code (3). …Nevermind
Jaywalker	The vehicle's front right sensors (1) detect a [pedestrian ahead who is walking into the street/large animal crossing the road ahead] (2). Based on their trajectory, the vehicle will brake and move to the left lane (3).	Cow in road	The vehicle's front right sensors (1) detect a [large animal/child] crossing the road ahead (2). Based on their trajectory the vehicle will brake and move to the left lane (3).
Fog	Please take over (1). The vehicle's light sensors (2) detect heavy [fog/rain] ahead (3).	Rain	Please take over (1). The vehicle's moisture sensors (2) detect heavy [rain/fog] ahead (3).

Bolded parts were presented in the high-reliability condition. *Italic* parts were presented in the low-reliability condition.

### 3.4. Procedure

The experimental procedure lasted at most 2 h. Upon arrival, participants were given a brief description of the study, and were asked to sign a consent form approved by the Virginia Tech Institutional Review Board. Participants were then given an explanation on the use of the driving simulator and performed a test drive to familiarize themselves with the device. This test drive only included manual driving and did not expose the driver to level 3 driving or any interaction with the IVAs to avoid any bias on the IVAs prior to the actual driving experiments. The primary purpose of the test drive was to evaluate participants’ motion sickness level on the driving simulator. To assess simulator sickness, participants were given a pre- and post- questionnaire both before and after the test drive to rate 17 symptoms of motion sickness. These were rated on a scale of 0 to 10, with 0 being “not at all” and 10 being “severe.” If simulator sickness was an issue, they were compensated and dismissed from the study. Two participants were dismissed due to simulator sickness. If not an issue, the demographics and emotional status surveys were completed. Participants were given sample paragraphs of a correlating emotional state (happy, angry, or neutral) and were given 12 min to write about a past positive or negative experience (happy or angry), or to write a detailed schedule of their previous day (neutral). This emotion induction method was validated in more than 20 previous emotional driving research studies (FakhrHosseini and Jeon, 2017). After completing the emotion induction task, participants repeated the emotional status survey as a manipulation check.

Before each simulated driving trial, participants were introduced to one of the robot agents. They were instructed that the robot may ask them to take over in certain situations, but they were allowed to choose not to. If the participant did take over the vehicle, they were required to hand control back when asked. Between each trial, participants were asked to complete the required questionnaires on their experience. Upon completion of both reliability conditions, participants completed a third emotional status questionnaire. They were then debriefed on the emotion induction to ensure that negative effects of emotions did not persist after the conclusion of the experiment.

### 3.5. Dependent measures

#### 3.5.1. Questionnaires

There were five categories of questionnaires used in the experiment. The first of these was a demographics survey, completed prior to starting the experiment. This asked participants about their age, gender, ownership of a driver’s license, years of driving experience, number of times driven per week, and the number of miles driven per year.

An emotional status questionnaire was completed three times throughout the experiment. The first was before the emotion induction, the second was immediately after emotion induction, and the last was after the completion of both trials. Participants were asked to rate fear, happiness, anger, depression, confusion, embarrassment, urgency, boredom, and relief on a seven-point Likert scale. These emotions were stated to be important to driving situations in a previous study (Jeon and Walker, 2011).

During two specified points in each scenario, the simulation was paused, and participants were asked to complete a Situational Awareness Global Assessment Technique (SAGAT) questionnaire to assess SA. The first trial was paused at a tunnel accident with a police car and semi-truck on the left, and then at an intersection where a car was stopped at a light. The second trial was paused inside of a tunnel, and then near a roadside accident with a police car and a man with a stroller on the right. The questionnaire consisted of five open-ended questions, divided into three levels. Level one questions, relating to perception, asked “What elements of interest do you see on the screen?” and “What vehicles did you notice around you?.” Level two questions, concerning comprehension of the event, asked “What do these elements tell you about the current situation?” and “What is currently happening in the scenario?.” Lastly, the level three question, concerning projection of future events, asked “What do you think will happen next?”

After each trial, participants completed two sets of questionnaires. The first was the NASA Task Load Index (TLX, Hart and Staveland, 1988) to measure subjective workload. The second set was a series of Subjective Judgment Ratings, which included the Godspeed questionnaire (Bartneck et al., 2008), the Social Presence scale (Harms and Biocca, 2004), the Robotic Social Aptitude scale (RoSAS, Carpinella et al., 2017), the Subjective Assessment of Speech System Interfaces scale (SASSI, Hone and Graham, 2000), the Trust in Automation scale (Jian et al., 2000), the Cognitive Trust scale (McAllister, 1995), and the Affective Trust scale (McAllister, 1995).

#### 3.5.2. Takeover performance

To compare subjective responses with actions, driving simulator recordings were taken of each trial. The compliance of the participants to the agent’s instructions was measured by whether there was a takeover of control. Additional performance measures included takeover reaction time, lane position, speed, longitudinal acceleration, lateral acceleration, steering wheel angle, jerk, and take over type (McDonald et al., 2019).

## 4. Results

All data were checked for sphericity and normality. When the sphericity assumption was violated, the Greenhouse–Geisser correction was applied. In a few cases, data were not normally distributed because some of the data came from the interval data (e.g., Likert-type data), but ANOVA was still applied to the data instead of the non-parametric analysis for the following reasons: (1) F-test (e.g., ANOVA or ANCOVA) is known as robust to violations of the interval data assumption and could be used to conduct statistical tests with no resulting bias (Carifio and Perla, 2007; Norman, 2010) and (2) non-parametric tests cannot show the interaction effects between variables, which we wanted to investigate in the present study.

### 4.1. Manipulation check

**Figure 2:**
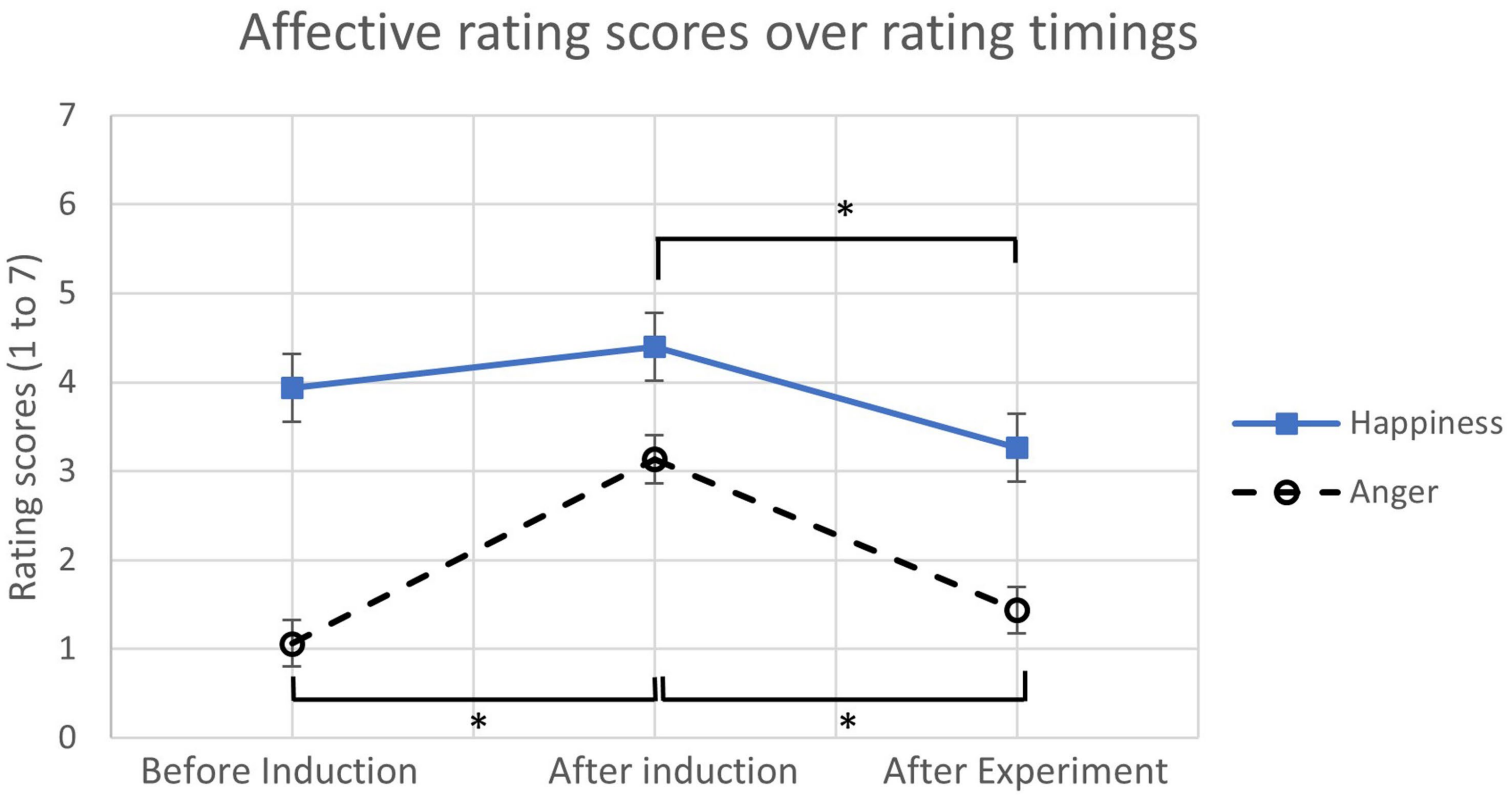
The average affective rating scores over different rating timings. (*: *p* < 0.0167, error bars represent standard errors).

The emotion induction results from the emotional status questionnaire were analyzed with a separate ANOVA for each emotion condition (happy, angry, and neutral). Only the corresponding emotional state was analyzed for each condition (e.g., happiness score for happiness condition, etc.). For the neutral condition, both happiness and anger scores were analyzed. Table 2; Figure 2 show the average of the affective rating scores over three timings: before induction, after induction, and after experiment. The results reported significant differences in the ratings scores of anger [*F*(2,28) = 18.41, *p* < 0.001, *η_p_^2^* = 0.55] and happiness [*F*(2,28) = 6.95, *p* < 0.01, *η_p_^2^* = 0.33], and no significant difference was found in the neutral conditions [happiness of neutral: *F*(2,30) = 1.70, *p* = 0.20; anger of neutral: *F*(2,30) = 1.18, *p* = 0.32]. With the Bonferroni correction (*α* = 0.0167), the average anger score from angry participants after induction was significantly higher than before induction (*p < 0*.01). In addition, the anger score after experiment was significantly lower than after induction (*p* < 0.01). For happy participants, the average happiness score after induction was numerically higher than before induction as expected, but it was not statistically significantly higher than before induction. However, the happiness score after experiment was significantly lower than after induction (*p* < 0.01) as well. Overall, for both happy and angry participants, the affective rating scores were subjectively higher after induction than before induction.

**Table 2:** Average affective rating scores over three timings. SD = ().

	Happiness	Anger	Happiness of neutral	Anger of neutral
Before induction	3.93	1.06	4.56	1.13
	(1.53)	(0.25)	(1.63)	(0.26)
After induction	4.40	3.13	3.93	1.07
	(1.68)	(1.60)	(1.87)	(0.34)
After experiment	3.27	1.44	3.94	1.24
	(1.16)	(0.89)	(1.75)	(0.75)

### 4.2. Situation awareness (SAGAT)

**Figure 3:**
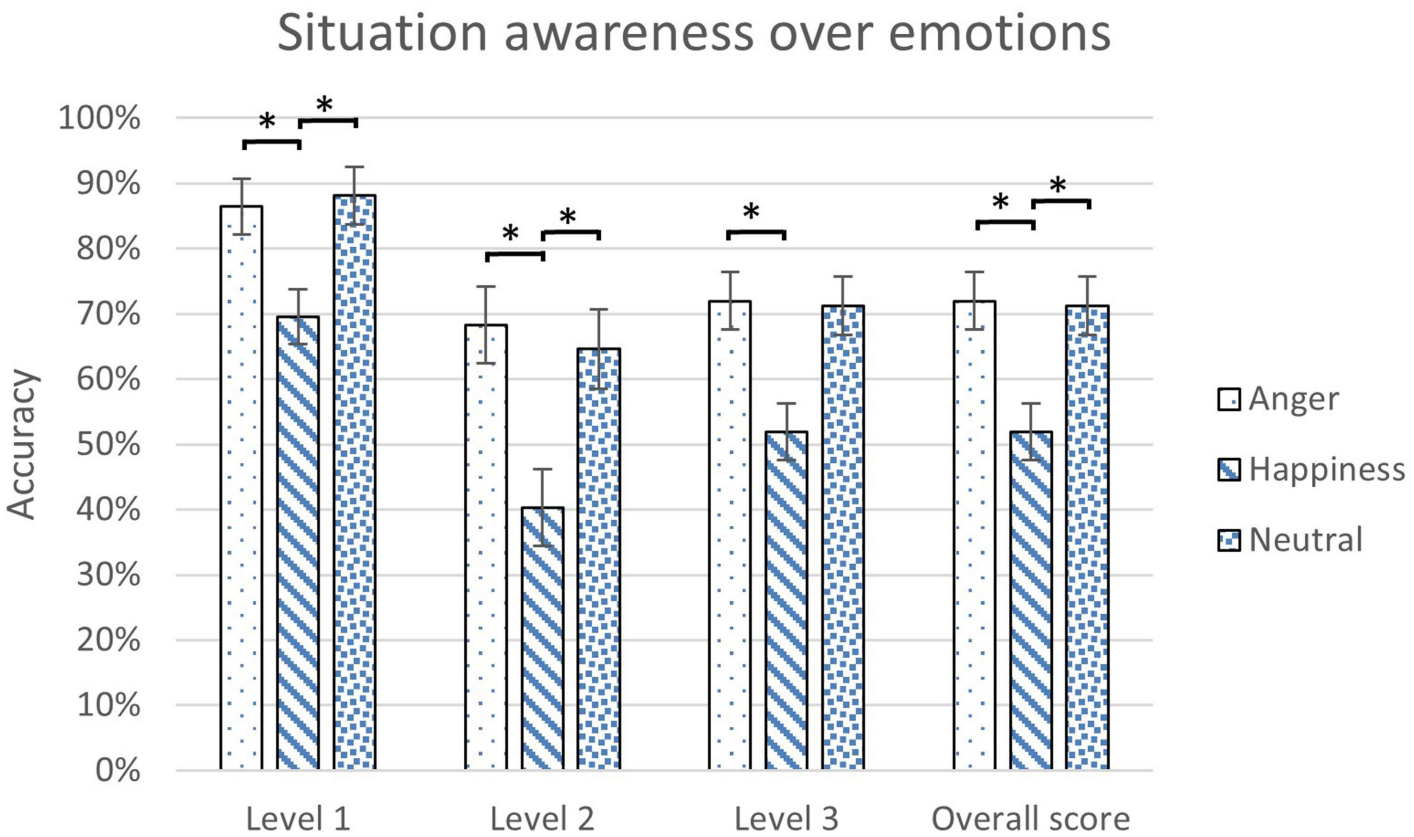
The scores of situation awareness over emotions for different levels of SA questions (*: *p* < 0.05, error bars represent standard errors).

A scoring rubric was made to grade participants’ responses in SAGAT. The average score of each participant was analyzed with 3 (Emotions) x 2 (Reliability Levels) mixed ANOVA for each condition. Emotions were found to have a main effect on SA (Table 3). No significant difference was found between the two reliability levels and in the interaction between emotions and reliability levels. According to least significant difference (LSD) *post hoc* tests, angry and neutral participants were found to have significantly higher SA scores than happy participants, especially for level 1 and level 2 questions (Figure 3).

**Table 3:** Statistics for SA (*p* < 0.05^*^)

Measures	Conditions		Statistics
Level 1	Main effect for emotions		*F*(2, 45) = 6.12, *p* < .01^*^, *η*_p_^2^ = 0.21
	Neutral *M* = 88.28%, SD = 0.16	Happiness *M* = 69.53%, SD = 0.22	*p* < 0.01^*^
	Anger *M* = 86.33%, SD = 0.19		*p* < 0.01^*^
	Main effect for reliability		*F*(1, 45) = 0.01, *p* = 0.93
	High reliability *M* = 81.25%, SD = 0.22	Low reliability *M* = 81.25%, SD = 0.19	N/A
Level 2	Main effect for emotions		*F*(2, 45) = 6.91, *p* < 0.01^*^, *η_p_^2^* = 0.24
	Anger *M* = 67.97%, SD = 0.27	Happiness *M* = 40.23%, SD = 0.30	*p* < .01^*^
	Neutral *M* = 64.84%, SD = 0.25		*p* < 0.01^*^
	Main effect for reliability		*F*(1, 45) = 0.26,*p* = 0.61
	High reliability *M* = 58.85%, SD = 0.30	Low reliability *M* = 56.51%, SD = 0.31	N/A
Level 3	Main effect for emotions		*F*(2, 45) = 2.92, *p* = 0.064^*^, *η*_p_^2^ = 0.16
	Anger *M* = 61.72%, SD = 0.22	Happiness *M* = 46.09%, SD = 0.27	*p* < .01^*^
	Main effect for reliability		*F*(1, 45) = 0.02, *p* = 0.89
	High reliability *M* = 56.25%, SD = 0.25	Low reliability *M* = 55.73%, SD = 0.24	N/A
Overall accuracy	Main effect for emotions		*F*(2, 45) = 6.96, *p* < 0.01^*^, η_p_^2^ = 0.24
	Anger *M* = 72.01%, SD = 0.19	Happiness *M* = 51.95%, SD = 0.23	*p* < 0.01^*^
	Neutral *M* = 71.09%, SD = 0.17		*p* < 0.01^*^
	Main effect for reliability		*F*(1, 45) = 0.13, *p* = 0.72
	High reliability *M* = 65.54%, SD = 0.23	Low reliability *M* = 64.50%, SD = 0.21	N/A

### 4.3. Subjective judgment ratings

The results of the subjective questionnaires (Godspeed, Social Presence, RoSAS, and SASSI) were analyzed with 3 (Emotions) x 2 (Reliability Levels) mixed ANOVA for each condition.

**Figure 4:**
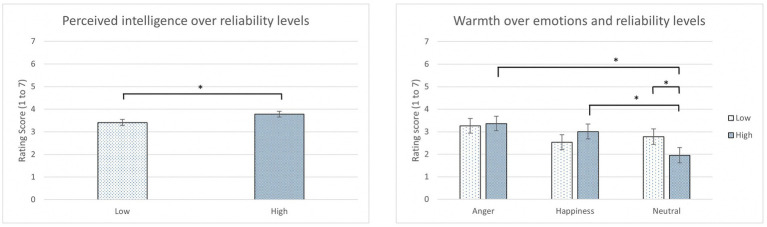
The average rating score of perceived intelligence over reliability levels (left) and the average rating score of warmth over emotions and reliability levels (right) (*: *p* < 0.05, error bars represent standard errors).

In the Godspeed and RoSAS questionnaires, Agent reliability was found to have a main effect on perceived intelligence [*F*(1,45) = 5.15, *p* = 0.03, *η_p_^2^* = 0.10; Table 4]. A significantly higher rating of perceived intelligence was found in the high reliability condition than the low reliability condition (*p* = 0.02; Figure 4 left).

**Table 4:** Statistics and Main effects for Godspeed, Social Presence, SASSI, RoSAS, Trust, and NASA-TLX items (*p* < 0.05^*^).

	Emotions					Reliability Levels			
	Mean (SD)			Significance		Mean (SD)		Significance	
	Angry	Happy	Neutral	*F*(2,45)	*p*	High	Low	*F*(1,45)	*p*
**Godspeed**									
Anthropomorphism	2.39(0.85)	2.12(0.76)	2.15(0.74)	0.80	0.46	2.28(0.81)	2.16(0.77)	0.95	0.33
Animacy	2.63(0.93)	2.34(0.68)	2.36(0.67)	0.81	0.45	2.49(0.76)	2.40(0.80)	1.12	0.30
Likeability	3.69(1.07)	3.21(0.80)	3.34(0.76)	1.91	0.16	3.49(0.89)	3.34(0.92)	0.95	0.34
Perceived Intelligence^*^	3.86(1.00)	3.29(0.83)	3.64(0.87)	2.65	0.08	3.78(0.81)	3.41(1.00)	5.15	0.03*
Perceived Safety	3.34(0.86)	3.17(0.73)	3.35(0.62)	0.44	0.65	3.24(0.83)	3.33(0.65)	0.66	0.42
**Social presence**									
Social presence	5.55(1.30)	5.34(1.61)	5.08(1.17)	0.54	0.59	5.34(1.47)	5.31(1.28)	0.03	0.86
**SASSI**									
System Response Accuracy	4.81(1.33)	4.59(0.95)	4.80(1.07)	0.26	0.77	4.91(1.07)	4.56(1.15)	3.81	0.06
System Likeability	4.78(1.25)	4.73(1.10)	4.67(0.91)	0.05	0.95	4.76(1.08)	4.69(1.10)	0.23	0.63
Cognitive Demand	3.31(1.34)	3.59(0.90)	3.19(0.97)	0.66	0.52	3.27(1.06)	3.46(1.12)	1.91	0.17
Annoyance	3.62(1.47)	3.48(0.81)	3.19(1.03)	0.72	0.49	3.43(1.25)	3.43(1.03)	0.00	0.98
Habitability	3.96(0.79)	3.81(0.65)	3.81(0.80)	0.31	0.74	3.90(0.76)	3.82(0.74)	0.38	0.54
Speed	4.39(1.26)	4.89(0.97)	4.33(1.32)	1.47	0.24	4.60(1.25)	4.47(1.17)	0.52	0.48
**RoSAS**									
Competence	4.71(1.30)	4.29(1.15)	4.66(1.21)	0.80	0.46	4.74(1.08)	4.37(1.34)	3.46	0.07
Warmth	3.26(1.54)	2.77(1.25)	2.48(1.24)	1.73	0.19	2.81(1.43)	2.87(1.32)	0.11	0.74
Discomfort	2.24(1.20)	2.01(0.82)	2.63(1.03)	2.16	0.13	2.27(1.05)	2.31(1.05)	0.05	0.82
**Trust**									
Trust in Automation	4.97(1.26)	4.61(1.01)	4.75(1.01)	0.55	0.58	4.89(1.03)	4.66(1.16)	2.10	0.15
Cognitive Trust	4.68(1.34)	4.38(0.94)	4.62(1.05)	0.51	0.61	4.75(0.95)	4.37(1.25)	3.90	0.05
Affective Trust	2.13(0.96)	2.69(1.42)	2.27(1.08)	1.14	0.33	2.39(1.13)	2.33(1.24)	0.19	0.66
**NASA-TLX**									
Physical Demand^*^	0.82(1.26)	3.47(4.20)	1.18(1.51)	5.07	0.01^*^	1.74(2.67)	1.90(3.13)	0.37	0.54
Mental Demand	10.59(7.53)	12.37(7.43)	11.76(7.41)	0.33	0.72	11.78(7.74)	11.37(7.15)	0.12	0.73
Temporal Demand	6.96(5.73)	10.18(9.30)	7.12(6.92)	1.14	0.33	8.55(7.90)	7.62(7.20)	0.95	0.33
Performance	6.22(4.01)	6.20(4.93)	7.58(5.70)	0.60	0.55	7.23(5.37)	6.10(4.41)	2.00	0.16
Effort	6.24(5.35)	7.80(5.82)	5.80(4.28)	0.82	0.45	6.58(4.93)	6.65(5.52)	0.01	0.92
Frustration	4.86(8.09)	3.17(3.87)	3.68(6.69)	0.38	0.70	4.13(7.46)	3.67(5.28)	0.24	0.63

There was also a statistically significant difference in warmth in the interaction between emotions and reliability levels [*F*(2, 45) *=* 4.33, *p* = 0.019*, η_p_^2^* = 0.16; Table 5]. According to LSD *post hoc* tests, angry and happy participants in the high reliability condition rated significantly higher scores of warmth than the neutral participants in the high reliability condition (*p =* 0.01 and *p* = 0.03, respectively). Also, neutral participants in the low reliability condition rated significantly higher scores of warmth than the neutral participants in the high reliability condition (*p =* 0.02; Figure 4 right). No significant difference was found between the two reliability levels and in the interaction between emotions and reliability levels in other items.

**Table 5:** Statistics and Interaction effect for Godspeed, Social Presence, SASSI, RoSAS, Trust, and NASA-TLX items (*p* < 0.05^*^).

	Emotions x reliability levels							
			Mean (SD)				Significance	
Measures	Angry, high	Angry, low	Happy, high	Happy, low	Neutral, high	Neutral, low	*F*(1,45)	*p*
**Godspeed**								
Anthropomorphism	2.54(0.86)	2.25(0.84)	2.26(0.81)	1.98(0.71)	2.04(0.72)	2.26(0.77)	2.06	0.14
Animacy	2.75(0.89)	2.51(0.98)	2.47(0.66)	2.22(0.71)	2.26(0.66)	2.47(0.69)	2.90	0.07
Likeability	3.74(1.18)	3.65(0.98)	3.39(0.68)	3.03(0.90)	3.34(0.72)	3.35(0.82)	0.56	0.58
Perceived intelligence	4.04(0.64)	3.68(1.26)	3.55(0.91)	3.03(0.68)	3.75(0.83)	3.54(0.93)	0.32	0.73
Perceived safety	3.13(1.00)	3.56(0.65)	3.21(0.80)	3.13(0.69)	3.40(0.69)	3.31(0.55)	2.43	0.10
Social presence								
Social presence	5.59(1.57)	5.51(1.01)	5.31(1.75)	5.36(1.51)	5.11(1.06)	5.05(1.31)	0.06	0.94
SASSI								
System response accuracy	4.97(1.21)	4.65(1.46)	4.82(0.98)	4.37(0.89)	4.93(1.09)	4.67(1.08)	0.11	0.90
System likeability	4.74(1.35)	4.82(1.18)	4.97(0.98)	4.49(1.18)	4.58(0.88)	4.76(0.97)	1.86	0.17
Cognitive demand	3.15(1.30)	3.48(1.41)	3.43(0.80)	3.75(0.98)	3.24(1.07)	3.15(0.90)	1.03	0.37
Annoyance	3.64(1.63)	3.60(1.33)	3.38(0.89)	3.59(0.75)	3.28(1.15)	3.11(0.91)	0.53	0.59
Habitability	3.91(0.82)	4.02(0.78)	3.94(0.70)	3.69(0.60)	3.86(0.79)	3.77(0.83)	0.67	0.52
Speed	4.50(1.37)	4.28(1.17)	4.88(0.99)	4.91(0.99)	4.44(1.39)	4.22(1.29)	0.20	0.82
RoSAS								
Competence	4.84(1.09)	4.57(1.50)	4.58(1.15)	4.00(1.12)	4.78(1.06)	4.54(1.37)	0.31	0.73
Warmth^*^	3.27(1.63)	3.24(1.49)	3.01(1.32)	2.53(1.17)	2.14(1.14)	2.83(1.28)	4.33	0.02^*^
Discomfort	2.19(1.26)	2.30(1.17)	2.06(0.92)	1.95(0.73)	2.57(0.95)	2.69(1.13)	0.20	0.82
**Trust**								
Trust in automation	5.09(1.19)	4.84(1.36)	4.81(0.88)	4.41(1.11)	4.77(1.02)	4.74(1.03)	0.48	0.62
Cognitive Trust	4.83(1.11)	4.53(1.56)	4.59(0.91)	4.16(0.95)	4.81(0.84)	4.42(1.22)	0.04	0.96
Affective trust	2.08(0.90)	2.17(1.04)	2.88(1.39)	2.50(1.48)	2.22(0.95)	2.33(1.22)	1.48	0.24
**NASA-TLX**								
Physical demand	0.77(1.26)	1.30(0.87)	3.92(3.10)	4.56(3.83)	1.53(1.35)	1.51(1.00)	1.44	0.25
Mental demand	10.73(7.43)	7.87(10.46)	8.06(12.77)	6.97(11.98)	8.08(11.85)	6.94(11.67)	0.02	0.98
Temporal demand	6.62(5.94)	5.68(7.29)	9.45(10.96)	9.39(9.40)	7.79(8.06)	6.03(6.19)	0.72	0.49
Performance	5.94(4.12)	4.00(6.50)	5.12(7.33)	4.62(5.06)	6.62(8.42)	4.68(6.75)	1.18	0.32
Effort	6.46(5.58)	5.28(6.02)	5.67(7.46)	6.14(8.15)	3.37(5.81)	5.14(5.79)	0.21	0.81
Frustration	4.71(8.80)	7.59(5.02)	4.37(3.15)	3.44(3.19)	8.72(4.54)	3.84(2.81)	0.47	0.63

No significance was found in either the Social Presence scales or SASSI scales.

### 4.4. Trust in automation, cognitive trust, and affective trust

**Figure 5:**
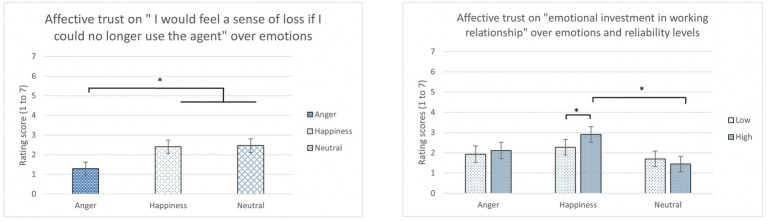
The average rating score of affective trust in “I would feel a sense of loss if I could no longer use the agent” over emotions (left) and “I would have to say that both the agent and I have made considerable emotional investment in our working relationship” over emotions and reliability levels (right) (*: *p* < 0.05, error bars represent standard errors).

The results of trust scales were analyzed with 3 (Emotions) x 2 (Reliability Levels) mixed ANOVA for each condition. Emotion was found to have a main effect on one of the affective trust item, “I would feel a sense of loss if I could no longer use the agent” [*F*(2, 45) = 4.01, *p* = 0.025, *η*_p_^2^ = 0.15]. Angry participants (*M* = 1.29, SD = 0.77) rated this affective trust item significantly lower than the neutral (*M* = 2.40, SD = 11.85; *p* = 0.02) and happy participants (*M* = 2.47, SD = 1.52; *p* = 0.02; Figure 5 left).

In addition, a significant difference was found in the interaction between emotions and reliability levels for another item in the affective trust, “I would have to say that both the agent and I have made considerable emotional investment in our working relationship” [*F*(2, 44) = 3.85, *p* = 0.029*, η_p_^2^* = 0.149]. According to LSD *post hoc* tests, participants who are in the happiness condition with high reliability (*M* = 2.91, *SD* = 1.71) reported significantly higher rating scores of Affective Trust on “emotional investment in working relationships” than those happy participants with low reliability (*M* = 2.28, *SD* = 1.91; *p* < 0.01) and those in the neutral condition with high reliability (*M* = 1.70, *SD* = 1.03; *p* = 0.01; Figure 5 right). No other significant differences were found for other trust items (Trust in Automation and Cognitive Trust) (Tables 4, 5).

### 4.5. Perceived workload

**Figure 6:**
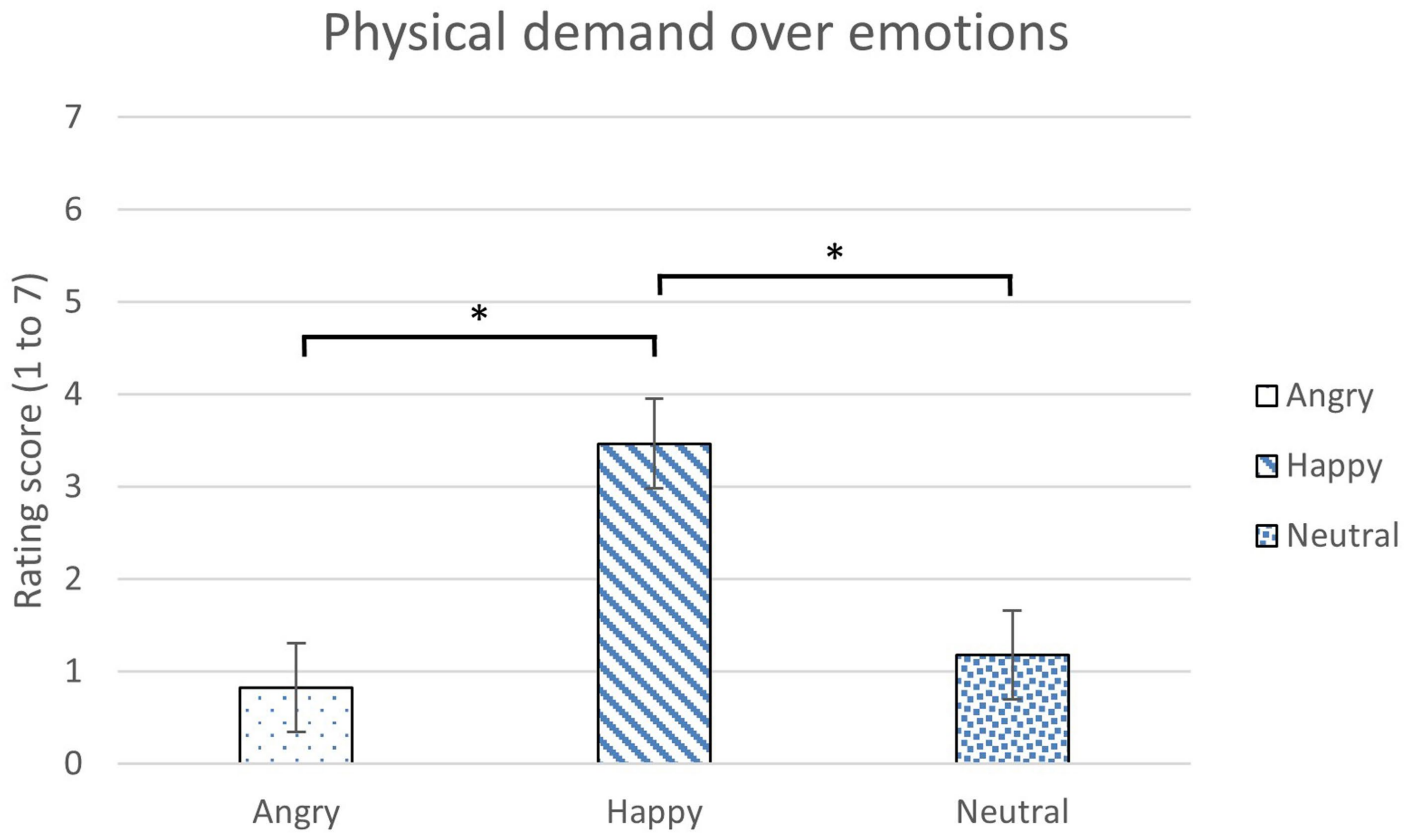
The average rating scores of perceived physical demand over emotions (*: *p* < 0.05, error bars represent standard errors).

The weighted perceived workload (NASA-TLX) over emotions was analyzed with 3 (Emotions) x 2 (Reliability Levels) mixed ANOVA for each condition. Emotion was found to have a main effect on physical demand (Table 5). No significant difference was found between reliability levels and in the interaction of emotions and reliability levels. Participants perceived the highest physical demand in the happiness condition (*p* = 0.01) than the anger and neutral conditions (*p = 0*.01; Figure 6). There was no significant difference found in the other dimensions of NASA-TLX.

### 4.6. Takeover performance

**Figure 7:**
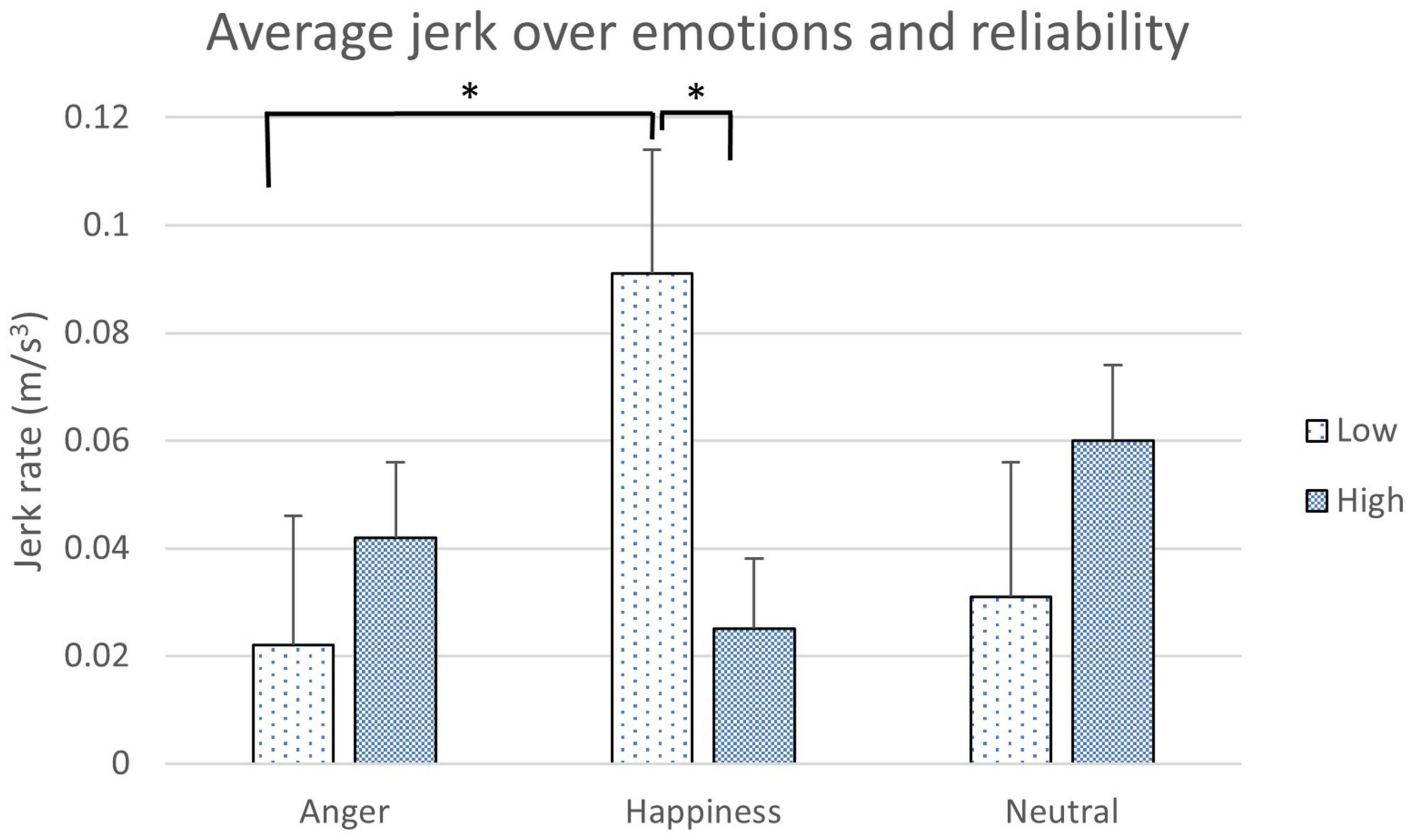
The jerk rate over emotions and reliability levels (*: *p* < 0.05, error bars represent standard errors).

The results of takeover performance were analyzed with 3 (Emotions) x 2 (Reliability Levels) mixed ANOVA for each condition. When a data value is over three standard deviations, it was considered as an outlier and removed from the data analysis. Two outliers were found in the takeover performance result; therefore, the sample size for this result was deducted by two (*N* = 46–2 = 44). There was a significant difference in the interaction between emotions and reliability levels for jerk in the takeover performance data (Table 6). Angry participants in the low reliability condition and happy participants in the high reliability condition had a significantly lower jerk rate than the happy participants in the low reliability condition (*p* = 0.05 and *p* = 0.01, respectively; Figure 7). The number of times participants complied with the agents were counted in each condition. No significant difference was found in other takeover performance items and compliance count (Tables 6, 7).

**Table 6:** Statistics and Interaction effect for takeover performance and compliance (*p* < 0.05^*^).

	Emotions x reliability levels							
			Mean (SD)				Significance	
Measures	Angry, high	Angry, low	Happy, high	Happy, low	Neutral, high	Neutral, low	*F*(1,45)	*p*
Take over time								
Take over time	3.44(1.76)	3.82(1.86)	3.70(1.88)	3.39(1.77)	3.40(1.98)	3.39(1.62)	0.36	0.70
Speed								
Maximum	23.77(5.49)	23.71(4.38)	23.64(5.26)	23.75(5.64)	24.78(6.47)	24.67(5.53)	0.01	0.98
Minimum	17.38(6.13)	17.98(4.66)	16.67(5.46)	16.83(5.72)	16.80(5.66)	17.80(5.24)	0.03	0.97
Average	20.56(5.36)	20.92(4.29)	20.16(4.86)	20.11(5.34)	20.87(5.12)	21.45(4.95)	0.09	0.92
Longitudinal acceleration								
Maximum	2.13(1.64)	1.91(1.21)	2.42(1.64)	2.40(1.59)	2.37(1.64)	2.02(1.26)	0.19	0.83
Minimum	–1.85(1.00)	–1.64(0.47)	–1.91(0.76)	–2.02(0.94)	–1.85(0.85)	–1.69(0.80)	1.00	0.37
Average	0.22(0.34)	0.20(0.26)	0.24(0.33)	0.29(0.34)	0.37(0.47)	0.29(0.34)	0.73	0.49
Lateral acceleration								
Maximum	0.92(0.58)	0.94(0.76)	1.12(1.65)	0.77(0.69)	0.82(0.79)	0.93(0.62)	1.71	0.19
Minimum	–1.71(1.13)	–1.70(1.16)	–1.75(1.60)	–1.54(1.03)	–1.71(1.27)	–1.78(1.20)	0.80	0.45
Average	–0.32(0.33)	–0.25(0.30)	–0.27(0.30)	–0.26(0.27)	–0.32(0.36)	–0.32(0.35)	0.69	0,51
Wheel angel								
Maximum	0.27(0.41)	0.21(0.19)	0.24(0.32)	0.20(0.26)	0.21(0.21)	0.22(0.21)	0.37	0.69
Jerk								
Maximum	81.96(37.45)	79.78(35.09)	93.71(40.01)	88.30(33.22)	89.80(34.85)	82.21(31.49)	0.09	0.92
Minimum	–57.29(29.35)	–55.15(15.00)	–66.21(30.40)	–64.12(24.79)	–60.93(30.27)	–60.10(30.29)	0.20	0.66
Average^*^	0.04(0.09)	0.02(0.04)	0.02(0.04)	0.08(0.21)	0.06(0.10)	0.04(0.10)	4.10	0.02^*^
Compliance								
Compliance count	3.43(0.85)	3.23(0.83)	3.00(0.52)	3.00(0.63)	3.19(0.66)	3.27(0.46)	0.33	0.72

**Table 7:** Statistics and Main effects for takeover performance and compliance (*p* < 0.05^*^).

	Emotions					Reliability levels			
	Mean (SD)			Significance		Mean (SD)		Significance	
	Angry	Happy	Neutral	*F*(2,43)	*p*	High	Low	*F*(1,43)	*p*
Take over time									
Take over time	3.63(1.81)	3.55(1.82)	3.39(1.79)	0.08	0.92	3.52(1.87)	3.52(1.74)	0.00	0.98
Speed									
Maximum	23.74(4.95)	23.69(5.42)	24.72(5.96)	0.57	0.57	24.04(5.71)	24.07(5.24)	0.01	0.90
Minimum	17.67(5.43)	16.75(5.56)	17.33(5.43)	0.86	0.44	16.93(5.70)	17.51(5.24)	0.61	0.44
Average	20.73(4.84)	20.14(5.07)	21.17(5.01)	0.94	0.40	20.51(5.07)	20.82(4.91)	0.23	0.63
Longitudinal acceleration									
Maximum	2.02(1.44)	2.41(1.61)	2.19(1.45)	1.29	0.24	2.31(1.63)	2.12(1.38)	0.72	0.40
Minimum	–1.75(0.79)	–1.96(0.85)	–1.76(0.82)	1.52	0.22	–1.87(0.86)	–1.79(0.79)	0.64	0.43
Average	0.21(0.30)	0.26(0.33)	0.33(0.41)	1.01	0.37	0.27(0.39)	0.26(0.32)	0.02	0.88
Lateral acceleration									
Maximum	0.93(0.67)	0.95(1.28)	0.88(0.70)	0.12	0.89	0.96(1.15)	0.88(0.69)	0.44	0.51
Minimum	–1.71(1.14)	–1.65(1.35)	–1.74(1.23)	0.11	0.90	–1.73(1.35)	–1.67(1.13)	0.14	0.71
Average	–0.29(0.31)	–0.26(0.29)	–0.32(0.35)	1.54	0.23	–0.30(0.33)	–0.28(0.31)	1.82	0.51
Wheel angel									
Maximum	0.24(0.32)	0.22(0.29)	0.21(0.21)	0.30	0.75	0.24(0.32)	0.21(0.22)	0.65	0.42
Jerk									
Maximum	80.90(36.09)	91.07(36.74)	85.77(33.13)	1.82	0.17	88.84(37.66)	83.66(33.10)	0.98	0.33
Minimum	–56.25(23.36)	–65.19(27.66)	–60.49(30.10)	1.75	0.19	–61.78(30.02)	–60.09(24.75)	0.20	0.66
Average	0.03(0.07)	0.05(0.15)	0.05(0.10)	1.08	0.35	0.04(0.08)	0.05(0.14)	0.04	0.84
Compliance									
Compliance count	3.33(0.83)	3.00(0.57)	3.23(0.56)	1.93	0.16	3.20(0.69)	2.89(0.64)	0.08	0.78

## 5. Discussion

To determine the impact of driver emotions and agent reliability levels on a Level 3 automated driving system, drivers’ responses from different measures including situation awareness, subjective perception, trust, perceived workload, and takeover performance were compared. Overall, the results showed that emotions play an important role in raising drivers’ attention to making observations in different driving situations; and the reliability of the agent impacts drivers’ perceived intelligence of the system. The interaction between the emotions and reliability levels has a distinctive impact for the perceived warmth of the system and a part of affective trust and takeover performance.

### 5.1. Emotion induction

Before looking at the results, deriving successful emotion inductions from participants was important. Participants were asked to write their past experience(s) with their assigned emotion, angry or happy, for 12 min. For neutral participants, they were asked to write events of their day in a chronological order. Both anger (significantly) and happiness (numerically) scores were higher after induction than before induction, which are consistent with the previous studies (e.g., Jeon et al., 2014a,b). Even though happy participants did not reveal a significantly higher score in the after induction than before induction, the previous studies showed the effectiveness of this emotion manipulation method (Jeon et al., 2014a,b). Happy participants’ emotional state might have been changed, but they might not be cognitively aware of that change. Different outcomes of their behaviors also supported this notion. The happiness score being significantly lower after the experiment might be due to the boringness and exhaustion (note that their physical demand was significantly higher than in other emotion conditions) from the driving scenarios. Anger scores after the experiment decreased dramatically to almost the same as before induction. These anger and happiness scores from before induction and after the experiment were similar to neutral participants’ scores (Figure 2; Table 2). Therefore, participants in the angry and happy conditions were “neutral” before the induction signifying the writing task successfully induced participants’ affective states to the designated emotion.

### 5.2. Situation awareness

Because in Level 3 automated vehicles, drivers may need to take over, maintaining situation awareness is vital. Participants in the angry (72.01%) and neutral states (71.09%) showed significantly higher situation awareness score than the participants in the happy state (51.95%) for all three levels (Table 3). Because happiness broadens the scope of attention (Derryberry and Tucker, 1994) or reduces the resources available for effortful processing (Mackie and Worth, 1989), it could have the participants neglect important details in the surroundings and divert their attention to other aspects (Jeon, 2015). In the study by Finucane (2011), negative emotions with a high arousal like anger enhanced selective attention because those emotions inhibit unrelated stimuli and narrow attentional focus (Easterbrook, 1959; Fredrickson, 1998). These findings might explain the SA result in the present study.

### 5.3. Subjective judgment ratings

The Godspeed ratings demonstrated part of the expected results. Participants reported higher perceived intelligence for the IVAs in the high reliability condition than in the low reliability condition (Table 4). When IVAs provided correct instructions that were related to the driving scenarios on time, participants might perceive the IVAs as responsive and robust, which were identified as the two characteristics that impact perceived intelligence (Krening and Feigh, 2018). This result could also serve as a manipulation check that participants noticed the difference in two reliability conditions.

Interestingly, in the RoSAS ratings, there was an interaction found between emotions and reliability levels in Warmth for the IVAs. Angry and happy participants perceived more warmth than the neutral participants in the high reliability conditions; in addition, neutral participants in the low reliability conditions also rated a higher warmth score than those in the high reliability conditions (Table 5). Warmth plays a major role in the impression formation process and positive interpersonal interactions, which also elicits emotions (Carpinella et al., 2017). This result might imply that when the IVAs provided accurate takeover instructions to the drivers with high reliability, the drivers who were induced to have emotions with a high arousal, such as angry and happy, were more likely to share the conversation and receive the information from the IVAs than the neutral drivers, which leads to a positive interaction during the driving scenarios (Berger, 2011). Meanwhile, neutral participants might perceive the IVAs’ mistakes as more tolerable and human-like and trust the robots more in the low reliability condition than in the high reliability condition because of the embodiment effect (Kontogiorgos et al., 2020). In the study by Kontogiorgos et al. (2020), the failures negatively impacted people’s perception of a smart speaker but not to a human-like embodied robot. With a sense of imperfection, people perceived the anthropomorphized robot as less machine like and more likeable (Salem et al., 2013). This finding could also explain why no significant results showed in the discomfort rating in different emotion and reliability conditions.

No significant difference was found for both Social Presence and SASSI scales, both of which measured participants’ subjective judgment toward the agent. Both IVAs are humanoid robots with a similar size, and they both used their own factory voices during the experiment. This might suggest that participants perceived similar social presence and efficiency and effectiveness of speech from both IVAs’ regardless of participants’ emotions and the reliability of the system in the present study.

### 5.4. Trust

No main effects were found in the Trust in Automation scale. It might imply that participants in different affective states still perceived relatively high trust (4.61 ~ 4.97) from the automated driving system or the robotic agents regardless of the emotion conditions. When working with an imperfect automation, people might benefit from calibrating their trust and adjusting attention to make better decisions (Parasuraman et al., 2000; Lee and See, 2004). Also, both cognitive trust and affective trust scales did not show the significant difference across emotion conditions. However, when closely looking into the data, there were significant differences found in a couple of items in the Affective Trust scale toward the IVAs. A main effect was found among the three emotions for “I would feel a sense of loss if I could no longer use the agent (McAllister, 1995).” Angry participants rated lower to this affective trust item than the happy and neutral participants. Because anger has high other responsibility and high self-control (Smith and Ellsworth, 1985), Dunn and Schweitzer (2005) suggested that anger would decrease trust. For instance, angry participants might have perceived IVAs to be responsible for the hazardous driving scenarios and considered they could control the situation for themselves without IVAs’ help. There was also an interaction between emotions and reliability for “I would have to say that both the agent and I have made considerable emotional investment in our working relationship (McAllister, 1995).” The result demonstrated that happy participants in high reliability conditions are more likely to build this affective trust in the cooperation with driving agents than participants in the low reliability conditions. It means that both happy (i.e., positive) emotion and high reliability matter to gain high affective trust. Based on our outcomes, we can cautiously infer that cognitive trust can be relatively easily formed compared to affective trust, but users’ positive emotions may promote to build affective trust, specifically when the system reliability is high. There is the possibility that when the previous studies (e.g., Hafızoğlu and Sen, 2018; Fahim et al., 2021) showed that happy emotions lead to high trust, it might be attributed to affective trust. However, little research has investigated the two constructs (cognitive and affective trust) separately as in our study. According to Lee and See (2004), trust is an affective response with some influences from analytic and analogical (i.e., cognitive) processes; in addition, the affective process of trust development has a greater impact on the analytic process side. More importantly, less cognitive demand is required to develop affective trust that links to the characteristics of agents and environments (Lee and See, 2004).

Even though the main effect of reliability in the cognitive trust score did not reach the traditionally significant level (*p* = 0.054; Table 4), the result might still imply that reliability levels showed the tendency to predict cognitive trust. However, this finding did not apply to affective trust because participants’ affective responses might be impacted more by the participants’ emotions than the reliability of the system.

### 5.5. Perceived workload

Happy participants perceived significantly higher perceived physical demand than both angry and neutral participants from the NASA-TLX result. This result might suggest that happiness motivated and engaged participants to perform the driving task which might require more physical effort (Joo and Lee, 2017). In the present study, participants experienced driving scenarios that were designed to contain dangerous driving situations that required takeovers, such as driving in a foggy weather and an unpredictable car accident ahead. A motivational theory suggested that the positive affect, such as happiness, might encourage the participants to work harder than other emotions in unpleasant situations to solve problems and maintain their positive state (Isen, 1987; Wegener et al., 1995; Jeon, 2015). In the study by Jeon et al. (2014b), happiness also had numerically higher scores in perceived workload than other emotions.

### 5.6. Takeover performance and compliance

An interaction between emotions and reliability levels was also found for the jerk rate. Jerky driving is considered as one of the aggressive actions that could cause driving accidents (Bagdadi and Várhelyi, 2011). Happy participants in the low reliability condition had a significantly higher jerk rate than the angry participants in the low reliability condition. Happy participants’ low situation awareness results partly explain this behavior in the two emotion conditions. Also, when happy participants are in the high reliability condition, they showed a significantly lower jerk rate than in the low reliability conditions. The perceived utility model by Blanchette and Richards (2010) suggested that emotions have complex impacts on decision-making and reasoning. In the low reliability condition, happy participants might perceive more frustration from the wrong instructions provided by the agents in the takeover events than the angry participants (Isen et al., 1988), which might lead to more jerky driving. Because happy participants performed more jerky driving, this could also explain why they perceived a higher physical workload than other emotions. Dealing with IVAs in the low reliability condition, angry participants might still insist their own decision-making power on driving due to their certainty and controllability (Ghasem-Aghaee et al., 2009). This result suggests that not only emotions contribute to driving behaviors but also having correct (reliable) takeover instructions is important, which can reduce the risk of aggressive actions in driving.

No significant differences were found in the number of compliances. The participants might have felt that takeover situations were all urgent and therefore, chose to switch to manual driving every time when the IVAs provided takeover instructions in an event. Also, the takeover instructions in the driving events might be very clear to handle, therefore, leading to similar compliance and other takeover performance items (takeover time, speed, longitudinal/lateral accelerations, and wheel angles).

### 5.7. Limitations and future work

Two participants quit the present study after the test drive because of motion sickness, which is very common in driving simulator studies (Kennedy and Frank, 1985). Participants seemed to over comply with the agent’s instructions, which might indicate that participants perceived a high level of reliability from the humanoid robots regardless of the designated reliability levels in emergency situations, such as takeover events (Robinette et al., 2016). Varying the level of urgency in the driving scenario would be of interest. Future studies could also incorporate the impact of different levels of anthropomorphism on drivers’ perceptions and trust on in-vehicle agents. The present study was conducted using a driving simulator, which may not fully reflect participant’s on-road takeover driving behaviors and their subjective responses from a real-life driving situation. Participants’ past experiences with automated vehicle might also influence their trust development with the system. However, the results could still approximate drivers’ behaviors on a CAV and provide insights on the design of future research studies. Although the happy state induction did not lead to statistically significant difference, we still noticed a numerical difference in participants’ happy states in the before and after induction. In future studies, physiological measurements can be included to compare with the subjective ratings of participants’ emotion states. Finally, the result of the present study might not represent the elder drivers or other populations’ responses to a Level 3 automated vehicle because the participants were all college students in the present study. These observations should be considered and improved in the future studies for generalization.

### 5.8. Design guidelines

Based on the overall outcomes from the present study and literature, we extracted practical guidelines for the design of conditionally automated vehicle (CAV) systems, and future research.

- In CAV system design, consider both drivers’ emotional states and an in-vehicle agent’s reliability because both factors impact drivers’ situation awareness, perceptions, trust, and takeover performance while driving.
- Given that emotional states can be dynamically changing, design the in-vehicle system to estimate drivers’ emotional state and adapt to it in real-time.
- When drivers are in a happy state, design the in-vehicle technologies and agents to mitigate drivers’ distraction and increase drivers’ situation awareness, while reducing their workload.
- Specifically, when drivers are in a happy state and the system reliability is low, design the system to improve drivers’ performance; for example, the system can provide real-time feedback about their inappropriate driving behaviors, such as jerk, so they can be aware of their negative driving performance.
- When drivers are in an angry state, design the in-vehicle technologies and agents to enhance their affective trust, awareness of their emotional state, and decision-making processes.
- In future research, vary different levels of urgency so that compliance can be differentiated between the different conditions.
- In future research, choose the variables in a sophisticated way to better disentangle cognitive trust and affective trust and investigate their effects on the interaction with the agent.

## 6. Conclusion

The purpose of the present study was to investigate how driver emotional states and CAV agent reliability influence situation awareness, subjective judgment, trust, workload, and takeover performance in Level 3 automated driving. The findings showed that SA was lower for happy participants compared to both angry and neutral participants (RQ1). Happy participants seemed more likely to be distracted from the takeover events in the present study. Most importantly, interactions between emotions and reliability levels occurred in subjective judgment (Warmth, Affective Trust), and performance (Jerk) (RQ2). Agents with high reliability were rated as having a higher perceived intelligence. In general, happiness with high reliability was contributed to those scales positively and benefited the driver’s behavior. However, the results also showed that low reliability can even negatively influence happy drivers. There was an absence of any significant results in the other trust scales and takeover compliances. This may imply that the influence of affective trust is independent of the influence on other trust forms. To conclude, the results imply that both positive emotion and high reliability are required in developing emotional relationships and trust with IVAs, which encourages positive driving behaviors.

## Data availability statement

The raw data supporting the conclusions of this article will be made available by the authors, without undue reservation.

## Ethics statement

The studies involving human participants were reviewed and approved by Virginia Tech Institutional Review Board. The patients/participants provided their written informed consent to participate in this study.

## Author contributions

SZ implementation of the driving scenario on the simulator, data collection, writing—original draft, and writing–review and editing. JD theoretical foundations and definition of research questions and hypotheses, formal analysis, writing–original draft, and writing—review and editing. ST implementation of the driving scenario on the simulator, data collection, and writing—review and editing. CS implementation of the driving scenario on the simulator, data collection, and writing—review and editing. MJ theoretical foundations and definition of research questions and hypotheses, conceptual design of the experiment, project administration, supervision, funding acquisition, and writing—review and editing. All authors contributed to the article and approved the submitted version.

## Funding

This study was supported by the Northrop Grumman Undergraduate Research Program.

## Conflict of interest

The authors declare that the research was conducted in the absence of any commercial or financial relationships that could be construed as a potential conflict of interest.

## Publisher’s note

All claims expressed in this article are solely those of the authors and do not necessarily represent those of their affiliated organizations, or those of the publisher, the editors and the reviewers. Any product that may be evaluated in this article, or claim that may be made by its manufacturer, is not guaranteed or endorsed by the publisher.
